# Mitochondria transcription and cancer

**DOI:** 10.1038/s41420-024-01926-3

**Published:** 2024-04-08

**Authors:** Tang Lei, Yu Rui, Zhou Xiaoshuang, Zhang Jinglan, Zhang Jihong

**Affiliations:** 1 Medical School, Kunming University of Science and Technology, Kunming, China; 2 Yunnan Province Clinical Research Center for Hematologic Disease, Kunming, China

**Keywords:** Oncogenes, Growth factor signalling, Mechanisms of disease

## Abstract

Mitochondria are major organelles involved in several processes related to energy supply, metabolism, and cell proliferation. The mitochondria function is transcriptionally regulated by mitochondria DNA (mtDNA), which encodes the key proteins in the electron transport chain that is indispensable for oxidative phosphorylation (OXPHOS). Mitochondrial transcriptional abnormalities are closely related to a variety of human diseases, such as cardiovascular diseases, and diabetes. The mitochondria transcription is regulated by the mtDNA, mitochondrial RNA polymerase (POLRMT), two transcription factors (TFAM and TF2BM), one transcription elongation (TEFM), and one known transcription termination factor (mTERFs). Dysregulation of these factors directly leads to altered expression of mtDNA in tumor cells, resulting in cellular metabolic reprogramming and mitochondrial dysfunction. This dysregulation plays a role in modulating tumor progression. Therefore, understanding the role of mitochondrial transcription in cancer can have implications for cancer diagnosis, prognosis, and treatment. Targeting mitochondrial transcription or related pathways may provide potential therapeutic strategies for cancer treatment. Additionally, assessing mitochondrial transcriptional profiles or biomarkers in cancer cells or patient samples may offer diagnostic or prognostic information.

## Facts

- Dysregulation of mitochondrial transcription factors affecting the metabolism, and proliferation of tumor cells
- The mitochondria transcription factor TFAM, POLRMT, TFBEM, and MTERFs is highly expressed in cancer and associated with tumor prognosis.
- However, the role of mitochondria transcription factors in cancer is controversial and needs further exploration.

## Open qusetions

- Whether mitochondrial transcription is associated with malignant progression of cancer?
- Whether dysregulation of mitochondrial transcription is associated with metabolic reorganization in cancer?
- What are the core regulators that connect the communication of mitochondria trancstption and nuclear?
- Are there differences between mitochondrial transcription dysregulation and cancer cells and cancer stem cells?
- How to target the mitochondrial transcription in cancer without affecting the normal cells?

## Introduction

Mitochondria are dynamic organelles involved in various cellular processes, including energy production, metabolism, and cell signaling [1, 2]. Proper regulation of mitochondrial functions is crucial for maintaining cellular homeostasis. Dysregulation of mitochondrial processes has been implicated in various diseases, including cancer [3–5].

Mitochondrial transcription plays a vital role in the biogenesis and maintenance of mtDNA, which encodes essential components of the OXPHOS system [6]. Transcription of mtDNA is carried out by a dedicated transcription machinery, consisting of mitochondrial RNA polymerase (POLRMT) and several transcription factors, including mitochondrial transcription factor A (TFAM), mitochondrial elongation factor (TEFM), mitochondrial transcription factor B2 (TFB2M) and mitochondrial termination factor (MTERF) [7].

In cancer cells, alterations in mtDNA and mitochondrial transcription have been observed, contributing to the rewiring of cellular metabolism and the acquisition of cancer-related phenotypes [8]. Aberrant mitochondrial transcription may result in dysregulated expression of OXPHOS components, leading to metabolic reprogramming, increased glycolysis, and decreased reliance on oxidative phosphorylation. Moreover, dysregulation of mitochondrial transcription factors, such as POLRMT, TFAM, TFB2M, and MTERFs, has been implicated in cancer development and progression [9–13]. These factors not only regulate mitochondrial gene expression but also play roles in DNA replication, repair, and maintenance of mitochondrial function. Alterations in the expression or activity of these transcription factors can affect mitochondrial integrity, promote mitochondrial dysfunction, and contribute to tumor initiation and progression.

Understanding the intricate relationship between mitochondrial transcription and cancer is essential for unraveling the underlying mechanisms driving tumorigenesis and identifying potential therapeutic targets. Targeting mitochondrial transcription and associated factors may offer new avenues for cancer treatment, aiming to disrupt cancer cell metabolism and restore mitochondrial functions. Further research in this field will provide valuable insights into the molecular mechanisms governing mitochondrial transcription in cancer and its potential implications for targeted therapies.

## Mitochondrial DNA transcription and its regulation

### Overview of the mitochondrial genome

**Fig. 1:**
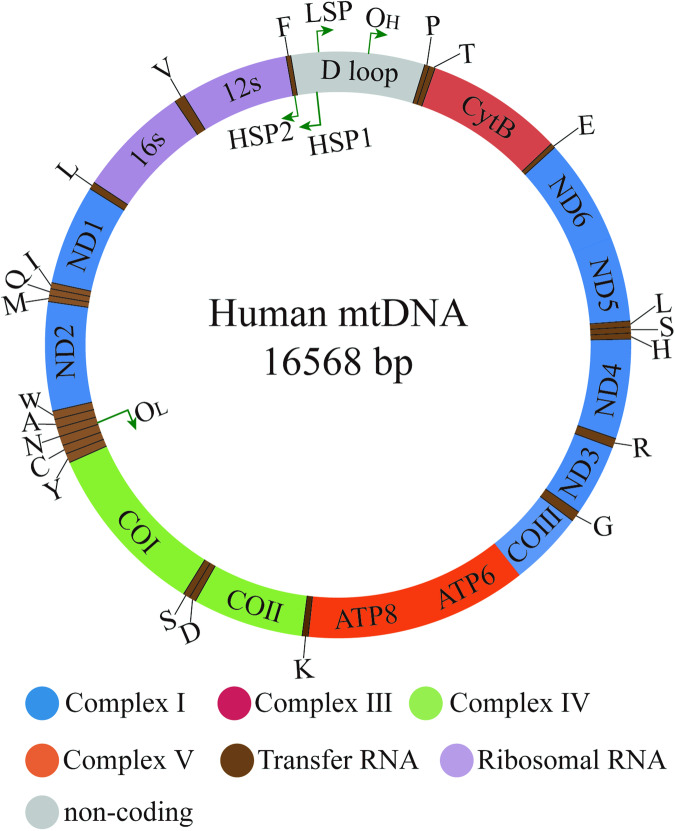
The structure of mitochondria genome. The mtDNA is a closed-circular, double-stranded DNA molecule comprising both high-replication and low-replication regions. It encodes 13 mitochondrial respiratory chain proteins, including complexes I (blue), III (red), IV (green), and V (orange), along with 22 tRNAs (brown) and 2 rRNAs (purple) involved in the translation of mitochondrial proteins.

The expression of mitochondrial genes is crucial for maintaining the homeostasis of eukaryotic cells. Mitochondria are unique in that their function is controlled by both the mitochondrial genome (mtDNA) and the nuclear genome (nDNA). Human mtDNA is a double-stranded closed circular molecule consisting of 16,569 base pairs, containing 37 genes encoding 13 subunits of oxidative respiratory chain proteins, 2 rRNAs, and 22 tRNAs (Fig. 1). The entire mitochondrial genome is transcribed into long polycistronic transcripts from both strands, which are separated into heavy strand (H) or light strand (L) based on their different buoyant densities in cesium chloride (CsCl) density gradient centrifugation. Replication and transcription of mtDNA begin at the non-coding region called the displacement loop region (D-loop) and are regulated by nuclear-encoded mitochondrial proteins. The mitochondrial D-loop region contains specific promoters for each strand of mtDNA transcription, namely the light strand promoter (LSP) and the heavy strand promoter (HSP), along with regulatory sequences controlling mtDNA replication. LSP is responsible for the transcription of 8 tRNA genes and the MT-ND6 gene. On the H strand, there is a dual promoter system. HSP1 generates transcripts containing tRNAPhe, tRNAVal, and 2 rRNAs (12S and 16S), while HSP2 produces transcripts that cover almost the entire genome.

### Mechanisms of mammalian mitochondrial transcription

**Fig. 2:**
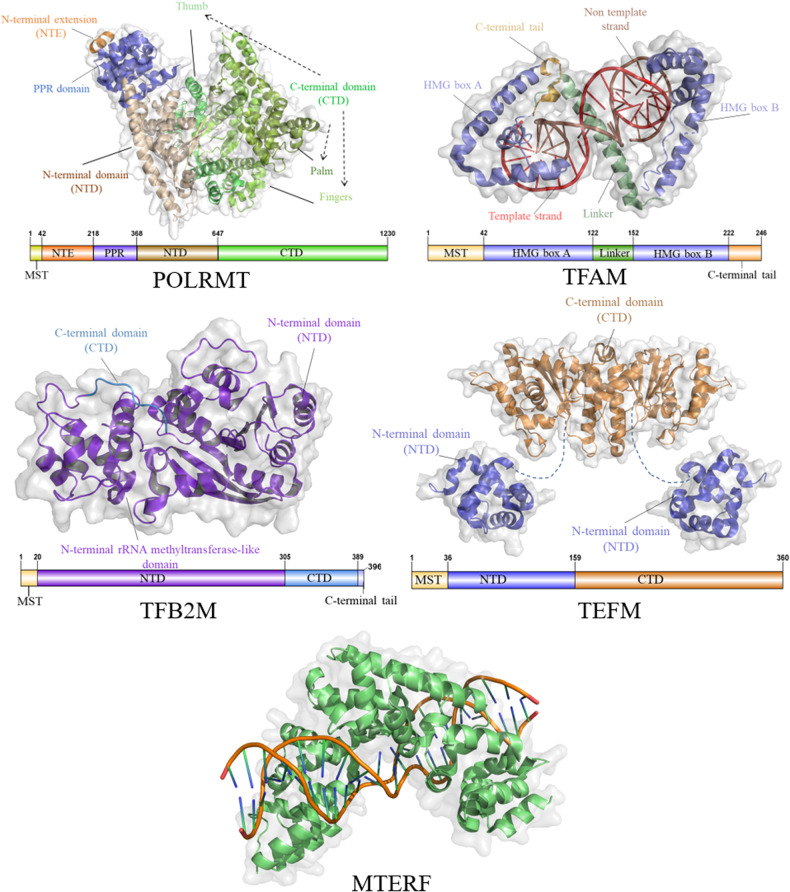
Structure of mitochondria transcription regulates factors. The POLRMT structure, visualized through the PDB file (PDBID: 3SPA), exhibits distinct domains: the mitochondrial targeting signal (MTS) in yellow, the N-terminal extension domain (NTE) in orange, the pentatricopeptide repeat domain (PPR) in blue, the N-terminal domain (NTD) in brown, and the C-terminal domain (CTD) in green. The TFAM structure, visualized via the PDB file (PDBID: 4NOD), includes the mitochondrial targeting signal (MTS) in yellow, the High Mobility Group Box A and B domains in blue, and the C-terminal domain (CTD) in orange. The TFB2M structure, visualized by the PDB file (PDBID: 6ERO), includes the mitochondrial targeting signal (MTS) depicted in yellow, the N-terminal rRNA methyltransferase-like domain (NTD) in purple, and the C-terminal domain (CTD) in blue. The TEFM structure, visualized using the PDB file (PDBID: 5OL8), showcases the mitochondrial targeting signal (MTS) in yellow, the N-terminal domain (NTD) in blue, and the C-terminal domain (CTD) in orange. The MTERF structure is represented by the PDB file (PDBID: 3MVA).

Mammalian mtDNA transcription occurs in the matrix and is primarily regulated by POLRMT, TFAM, TFB2M, and mTERFs (Fig. 2), which involves three main steps: initiation, elongation, and termination.

#### Mitochondrial transcription initiation

The transcription process of mammalian mtDNA is primarily driven by POLRMT. POLRMT is a DNA-dependent RNA polymerase and belongs to the T7-like RNA polymerase family. It has a total length of 1230 amino acids (~134 kDa) and consists of four structural domains: the N-terminal extension, a pentatricopeptide repeat (PPR) domain, the N-terminal domain, and the C-terminal domain (Fig. 2) [14]. The N-terminal extension domain is located at the beginning of the protein and may play a role in regulating the activity of POLRMT [15]. The pentatricopeptide repeat (PPR) domain is involved in protein-protein interactions and may contribute to the stability and specificity of POLRMT binding to mtDNA promoters [16]. The N-terminal domain is responsible for DNA binding, and it recognizes specific sequences within the mtDNA promoter regions. This domain enables POLRMT to initiate transcription at the appropriate sites [17]. The C-terminal domain is involved in RNA polymerization and catalyzes the synthesis of RNA using the DNA template. It ensures accurate and efficient elongation of the nascent RNA transcript [7].

**Fig. 3:**
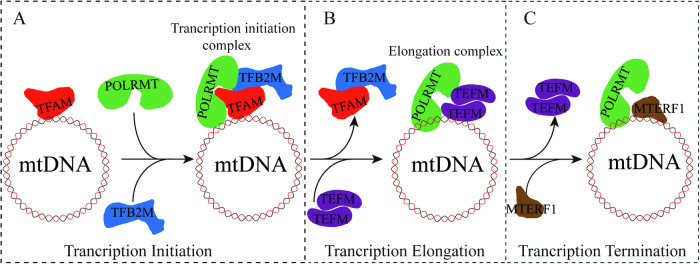
Model of mammalian mitochondrial transcription. **A** The initiation complex formed by POLRMT, TFAM and TFB2M assembles in the promoter region. **B** TFAM and TFB2M disengage from the initiation complex and TEFM binds POLRMT to form the elongation complex. **C** Transcription from LSP is terminated by MTERF1 that binds the mt-Tl1 sequence.

Although POLRMT is the key catalytic component in the transcription process of mtDNA, it does not possess the ability to independently recognize the L-strand (LSP) and H-strand (HSP) promoters. It requires the assistance of two factors, TFAM and TFB2M, to initiate the formation of the transcription initiation complex [18, 19]. During transcription initiation, TFAM first recognizes the promoter region and binds to mtDNA, causing it to bend into a U-shaped conformation, which activates transcription [20]. Simultaneously, POLRMT is recruited by TFAM, where its N-terminal extension (NTE) recognizes and interacts with both mtDNA and TFAM. Subsequently, TFB2M enters the transcription initiation complex, leading to the unwinding of the promoter region of mtDNA, forming a transcription initiation bubble [14, 21] (Fig. 3A). Using mtDNA as a template, POLRMT synthesizes a short RNA chain of approximately 25 nucleotides as the primer for transcription initiation. This short RNA chain serves as the starting point for the assembly of the transcription machinery and subsequent elongation of the RNA transcript [22].

#### Transcription elongation

During the elongation stage, TFAM and TFB2M dissociate from the POLRMR-mtDNA complex, allowing transcription elongation to proceed [23], POLRMT requires an additional transcription elongation factor called TEFM [24]. TEFM consists with four structural domains: an N-terminal mitochondrial targeting sequence, a helix-hairpin-helix (HhH) domain, an intermediate linker domain, and a C-terminal RuvC-like resolvase fold domain [25]. TEFM forms a homodimer through interactions between the C-terminal fold domains. (Fig. 2) It then interacts with the C-terminal catalytic domain of POLRMT and participates in the transcription elongation process, regulating mtDNA replication and transcription. These interactions contribute to the formation of a “sliding clamp” with downstream DNA, thereby enhancing the progression of the transcription elongation complex [7, 23, 25, 26]. TEFM binds to POLRMT and stabilizes the elongation complex, allowing POLRMT to continue transcription through the conserved sequence block II (CSBII), resulting in the generation of longer transcripts (Fig. 3B) [27].

#### Transcription termination

Transcription of mtDNA terminates upon specific binding of the mitochondrial termination factor (MTERF) to mtDNA (Fig. 3C) [25]. MTERF is a class of highly conserved mitochondrial DNA-binding proteins encoded by nuclear genes, including four different subtypes [28]. Among them, MTERF1 is the only known transcription termination factor, MTERF1 comprises 342 amino acids and has two independent DNA-binding domains and three leucine zipper-like structures, acting on mtDNA as a monomer. The DNA-binding domains of MTERF1 contain eight repeated mTERF motifs, each consisting of two alpha helices (Fig. 2). Hydrophobic interactions between adjacent motifs stabilize the protein but induce unwinding and base flipping of the targeted mtDNA sequence [29]. Studies have shown that MTERF1 inhibits the affinity between POLRMT and the template by specifically binding to a 28-nt sequence located on mtDNA that corresponds to the 16S rRNA gene and the tRNA ^Leu (UUR)^ gene. This leads to premature termination of heavy chain transcription [30]. Recent research suggests that recombinant MTERF can bind to the tRNA ^Leu (UUR)^ site and terminate transcription in a bidirectional manner. The efficiency of transcription termination is higher when POLRMT transcribes in the direction of the L-strand [31].

## Dysregulation of mitochondrial transcription affects tumor progression

**Fig. 4:**
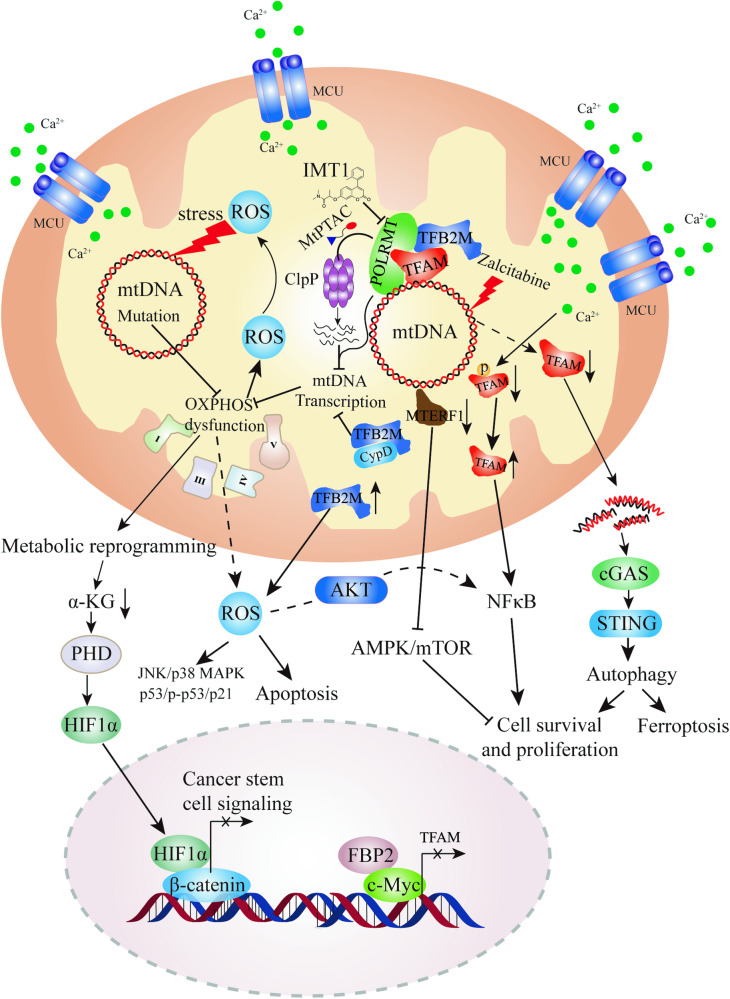
Mechanism of mitochondria transcription in cancer progression and the regulation of mitochondria transcription for cancer treatment. Mitochondrial DNA mutations and inhibition of mitochondrial transcription lead to damage in oxidative phosphorylation, promoting ROS release and modulating signaling pathways such as MAPK/mTOR, AKT, and NF-κB signaling pathways, thereby affecting the proliferation and apoptosis of tumor cells. Silencing of TFAM expression in mitochondria induces metabolic reprogramming in tumor cells, leading to the release of α-KG, which downregulates β-catenin transcriptional activity, suppressing tumor stem cell signaling. Additionally, inhibiting TFAM expression mediates the release of mitochondrial DNA into the cytoplasm, activating the cGAS-STING pathway and promoting autophagy-dependent ferroptosis. Small molecule compounds IMT1 and MtPTAC target POLRMT to inhibit mitochondrial transcription, suppressing mitochondrial OXPHOS and inhibiting tumor cell proliferation.

Mitochondria occupy a critical position in the cellular metabolic network, and the expression of mtDNA is closely associated with nearly all cellular metabolic activities [32]. Transcriptional regulation of mtDNA is particularly crucial. Mitochondrial transcription directly or indirectly affects mitochondrial respiration and cellular metabolism [33, 34]. Mitochondrial transcription dysregulation has been implicated in various diseases [35–39], including cancer [9, 40–45]. In cancer cells, alterations in mitochondrial transcription can contribute to tumor development and progression [9, 40, 41]. Dysregulated mitochondrial transcription can result in aberrant expression of mitochondrial genes involved in energy production, apoptosis, and other cellular processes [33, 46–48]. These changes may impact the metabolic reprogramming of cancer cells, promoting their survival, proliferation, and resistance to therapy (Fig. 4). Thus, understanding the role of mitochondrial transcription in cancer can have implications for cancer diagnosis, prognosis, and treatment.

### mtDNA mutation and cancer

During tumorigenesis and the process of tumor evolution, genetic mutations occur alongside sustained genomic instability, and this is also evident in the mitochondrial genome [49]. The human mitochondrial genome is akin to a polycistronic transcript, and even the D-loop, which can be seen as an intron, is an indispensable component for the transcription and replication of mitochondrial genes. Hence, any erroneous mutations in the mitochondrial genome have a profound impact on the biological function of mitochondria. Numerous studies have shown that variations in mtDNA are detected in over 50% of tumors [50]. The alterations in mtDNA copy number and mutations are closely associated with tumor occurrence, development, treatment, prognosis [51–59]. mtDNA mutations have also been found to increase the risk of thyroid carcinoma [60], breast cancer [61], lung adenocarcinoma [62, 63], and acute myeloid leukemia [64, 65] development. Anna L. and colleagues discovered that the accumulation of mtDNA mutations in aging colonic epithelial cells induces metabolic reprogramming due to OXPHOS defects, thereby accelerating intestinal cancer development functionally [66]. Exon sequencing of 70 cases of Hürthle Cell Carcinoma revealed that over half of the patients exhibited mtDNA loss-of-function mutation (LOF) and missense mutations in the gene encoding the electron transport chain complex I subunit, with these mutations predominantly occurring in highly conserved regions. These mutations can lead to defects in mitochondrial complex I and serve as a driving factor in the early formation of clones during tumor development [60]. Mutations in mtDNA can also lead to impaired mitochondrial respiratory chain function and potentially stimulate the production of reactive oxygen species (ROS). ROS, in turn, can induce mutations in genes involved in regulating cell replication, including oncogenes and tumor suppressor genes, leading to the development of cancer. Additionally, mild mtDNA mutations may participate in metabolic remodeling and potentially impact tumor progression, conferring the ability for tumor metastasis [67]. Although mutations in mtDNA are directly associated with tumors, some studies suggest that mtDNA mutations may be “passengers” rather than “drivers” in the process of tumorigenesis [68–70]. For example, in colorectal cancer (CRC), despite the general increase in oxidative metabolism in CRC cells, the somatic mutations in mtDNA within CRC tissues are not closely associated with mitochondrial biogenesis, oxidative metabolism, and clinical progression [70]. However, the biological consequences of mtDNA variations are highly dependent on the environment, including tissue type, tumor microenvironment, and nDNA genotype. Moreover, there is a lack of key experimental methods to assess the pathogenicity of specific mtDNA variations. Therefore, evaluating the pathogenic impact of mtDNA variations requires more stringent genetic and biochemical analyses [71].

### mtDNA as potential diagnostic, prognostic biomarkers and therapy target

Mutations and changes in copy number of mtDNA are among the most common features in tumor progression, making mtDNA as a potential molecular tool for early tumor detection. Compared to nuclear DNA (nDNA), mtDNA has unique advantages. It is shorter in length and has a simpler structure, making whole-genome scanning of mtDNA more convenient and efficient than using nDNA. Moreover, the higher abundance of mtDNA molecules significantly enhances their capacity as biosensors, improving sensitivity and accuracy in detecting rare malignant cells or even individual cells [72, 73]. For example, Willemina et al. [74] demonstrated the frequent occurrence of mtDNA D310 single nucleotide repeats mutations in several types of tumors, including breast, head and neck, lung, colorectal, and skin tumors. For these patients, D310 mutations could be used to determine the clonal relationship between their multiple tumors. Their research also suggested that D310 is a reliable marker for tracking cancer cell clonal expansion, and D310 mutations may help in clinically determining the clonal formation capability of synchronous or metachronous tumors. Furthermore, the discovery of circulating cell-free mitochondrial DNA (ccf mtDNA) in the plasma and serum of cancer patients has also sparked interest in its diagnostic value [75, 76].

In addition to serving as a potential early diagnostic marker for cancer, mtDNA can also be targeted for effective tumor immunotherapy. Ionizing radiation-induced mtDNA breaks lead to the release of mitochondrial RNA into the cytoplasm through BAX and BAK mediation, triggering a robust RIG-I-MAVS-dependent immune response, thereby enhancing tumor innate immune surveillance [22]. Similarly, inhibition of Ataxia telangiectasia mutated (ATM) promotes the release of tumor mtDNA into the cytoplasm, activating the cGAS/STING signaling pathway-mediated type I interferon immune response, facilitating the infiltration of CD8 + T cells in the tumor microenvironment, and suppressing tumor resistance to PD-1 therapy [23].

### Role of POLRMT and cancer

POLRMT is a core component of the mitochondrial transcription machinery and is closely associated with tumor progression. Studies have shown that increased expression of POLRMT is observed in lung cancer [9], osteosarcoma [10], squamous cell carcinoma [41], endometrial cancer [40], and acute myeloid leukemia [77, 78], and it can promote tumor cell proliferation, invasion, and migration abilities. The high expression of POLRMT is directly associated with poor prognosis in cancer patients [9, 41, 79]. Furthermore, POLRMT have been found to increase the risk of oral cancer and leukoplakia, possibly by modulating the synthesis and activity of enzymes [80]. These findings suggest that POLRMT may be a key gene involved in promoting cancer progression. However, the underlying molecular mechanisms by which POLRMT regulates tumor progression are not yet fully understood. Ahmed et al. suggest that high expression of POLRMT in breast cancer promotes mitochondrial biogenesis, providing the energy necessary for sustaining cell proliferation and conferring tolerance to autophagy, ultimately promoting tumor growth [81]. Recently, a study evaluated the role of POLRMT expression and function in lung adenocarcinoma using multi-omics analysis. Gene set enrichment analysis revealed a positive correlation between POLRMT expression and the expression of downstream target genes of the Wnt/β-catenin signaling [82]. Furthermore, POLRMT expression was positively associated with immune-suppressive genes, thereby affecting immune infiltration [82].

### Target POLRMT for cancer treatment

The significant role of POLRMT in cancer makes it an emerging therapeutic target for cancer treatment [83]. The small molecule compound IMT1 is the first allosteric inhibitor targeting POLRMT. IMT1 inhibits the transcriptional activity of POLRMT, thereby suppressing the synthesis of mitochondrial OXPHOS proteins and causing an energy crisis in tumor cells, leading to inhibition of tumor proliferation. Importantly, IMT1 exhibits broad-spectrum anti-tumor effects without significant side effects in vivo [84]. Li et al. optimized the structure of IMT1 and obtained more potent derivatives with enhanced anti-tumor activity both in vitro and in vivo [85]. Wang et al. developed a mitochondrial protease targeting chimera (MtPTAC) by linking the POLRMT-targeting inhibitor IMT1 with the mitochondrial protease ClpP. They found that MtPTAC effectively promotes the degradation of POLRMT mediated by ClpP in mitochondria and demonstrates its promising anti-tumor activity in vitro and in vivo [86]. This study confirms the findings proposed by Daglish et al. [87]. Additionally, knockdown the POLRMT expression in vitro and in vivo also effectively inhibits tumor cell proliferation and migration [9, 10, 40, 41].

### Role of TFAM in cancer

In addition to maintaining functionality in normal cells, TFAM also plays a crucial role in the tumor process. However, in different tumor cells, the expression of TFAM may exhibit two opposing functions.

On one hand, higher expression TFAM has been found to have a promoting role in the development and progression of certain tumors. TFAM is upregulated in prostate cancer [88], glioma [89], and breast cancer [90], and showing a positive correlation with poor patient prognosis. TFAM is also upregulated in drug-resistant liver cancer cells, and inhibiting TFAM expression enhances the sensitivity of resistant cells to chemotherapy [11]. Deletion of the TFAM gene leads to mitochondrial dysfunction and reduces tumor incidence in a Kras-driven lung cancer mouse model [91]. Colorectal cancer cells increase Ca^2+^ uptake, which activates phosphodiesterase 2 (PDE2) and inhibits the activity of mitochondrial protein kinase A (PKA), leading to the stabilization of TFAM accumulation in mitochondria and promoting colorectal cancer cell proliferation [92]. Similarly, Liu et al. found that increased mitochondrial Ca^2+^ uptake upregulates TFAM expression, promoting mitochondrial biogenesis and increasing mitochondrial ROS production, subsequently activating the NF-κB signaling pathway, and accelerating the growth of colorectal cancer [93]. Mutation in TFAM also promotes cell proliferation rate and enhances tumorigenicity in xenograft models [56]. Silencing TFAM expression in colorectal cancer mediates metabolic reprogramming, inducing α-ketoglutarate (α-KG)-mediated inhibition of the Wnt/β-catenin signaling pathway, thus suppressing tumor initiation [94]. Knockdown of TFAM expression in non-small cell lung cancer inhibits tumor cell proliferation by activating the ROS-mediated JNK/p38MAPK signaling pathway and reducing cellular bioenergetic production [95]. Decreased expression of TFAM induces G1/S phase arrest in tumor cells, enhances the interaction between p53 and MDM2, resulting in decreased expression of p53 and its downstream target gene TIGAR, and increases the sensitivity of tumor cells to ionizing radiation [96].

On the other hand, some studies suggest that downregulation of TFAM expression may be associated with tumor development. The low expression of PGC1a and TFAM has been proposed as predictive markers for chemoresistance in the epithelial ovarian cancer subtype [97]. Inhibiting TFAM expression in ovarian cancer promotes tolerance of chemotherapy drugs, attenuating mtROS and cisplatin-induced apoptosis [98]. Decreased TFAM expression promotes the release of mtDNA into the cytoplasm, inducing cytoplasmic mtDNA stress and activating the cGAS-STING signaling pathway, which stimulates autophagy and promotes esophageal squamous cell carcinoma growth [94]. Knockdown of TFAM expression in breast cancer reduces the mtDNA copy numbers and activates Calcineurin-mediated mitochondrial retrograde signaling, upregulates mesenchymal gene expression to induce EMT, and generates cancer stem cells [99]. In head and neck cancer, TFAM and mtDNA expression are significantly decreased in tumors compared to normal counterparts and negatively correlated with disease progression [100]. TFAM silencing enhances cell growth and chemoresistance, while significant reversal of these phenotypic changes is observed with increased TFAM expression, which is mechanistically associated with cell metabolic reprogramming and the ERK1/2-Akt-mTORC-S6 signaling pathway [100].

### Target TFAM in cancer

The significant role of TFAM in tumors makes it a potential therapeutic target for cancer treatment, with modulating TFAM expression or activity holding promise for developing novel strategies to selectively target cancer cells. The natural small molecule compound: 2,3,5,6-tetramethylpyrazine (TMP) interacts with TFAM and prevent Lon-mediated degradation of TFAM, leading to TFAM accumulation and subsequent up-regulation of mtDNA content in cells [101]. The antiviral drug zidovudine induces TFAM degradation, leading to mtDNA depletion and oxidative damage, and triggers autophagy-dependent ferroptosis, resulting in cell death in primary and immortalized human pancreatic cancer cells [102]. Melatonin reduces TFAM expression in human glioblastoma cell line U87MG, disrupting mtDNA expression and causing cell death due to increased ROS production and mitochondrial damage [103]. The first-in-class imipridone molecule ONC201 exerts its anticancer effects by activating mitochondrial protease ClpP to degrade TFAM expression [104]. In addition to small molecule compounds that modulate the expression of TFAM, the oncogene c-Myc promotes nuclear transcription of TFAM, facilitating sarcoma growth, while Fructose-1,6-Bisphosphatase 2 (FBP2) overexpression inhibits c-Myc-mediated transcriptional regulation of TFAM [105]. Ionizing radiation enhances TFAM mRNA stability by promoting RNA-binding protein HuR binding through the ataxia-telangiectasia mutated kinase/p38 (ATM/p38) signaling pathway, leading to increased TFAM expression and activation of radiation-induced damage repair [106]. Inhibition of TFAM is proposed as a therapeutic strategy to enhance cellular sensitivity to ionizing radiation [106]. Huang et al. demonstrated that ROS-mediated mitochondrial cell death can be rescued through TFAM complementation, suggesting that TFAM may act as an antagonist of ferroptotic cell death [107].

Due to the heterogeneity and complexity of tumors, TFAM may exhibit different functions and regulatory mechanisms in different types of cancer. For example, TFAM plays a dual role in mouse intestinal tissue [108]. TFAM overexpression in normal tissue can inhibit tumor development; however, TFAM expression is upregulated in colitis-associated cancer (CAC) tissues and contributes to cell growth [108]. Therefore, further research is needed to better understand the specific roles and molecular mechanisms of TFAM in specific types of cancer. Overall, the role of TFAM in tumor development and progression is a complex area that requires further investigation. Gaining a deeper understanding of TFAM’s functions and regulatory mechanisms in different types of tumors may contribute to the development of novel therapeutic strategies and the prediction of tumor progression and prognosis.

### Role of TFB2M in cancer

TFB2M also plays a crucial role in the transcription of mitochondrial DNA (mtDNA) and maintaining mitochondrial function. While there is limited specific information available on the role of TFB2M in cancer, dysregulation of mitochondrial transcription factors, including TFB2M, has been implicated in various types of cancer.

Specifically, the overexpression of TFB2M in ovarian cancer is negatively correlated with the survival rate of ovarian cancer patients and moderately positively correlated with tumor-associated macrophage (TAM) infiltration [12]. TFB2M overexpression is also associated with increased extracellular mtDNA and elevated IL-6 expression in ovarian cancer cells and promote the infiltration of M2 macrophages through the cytoplasmic mtDNA/TLR9/NF-κB/IL-6 pathway [12]. In hepatocellular carcinoma (HCC), the overexpression of TFB2M is associated with abnormal activation of the ROS-Akt-NF-κB signaling pathway, promoting tumor growth and metastasis. Inhibiting TFB2M may help suppress the abnormal activation of the ROS-Akt-NF-κB signaling pathway and slow down or prevent the development of HCC [109]. Furthermore, the overexpression of TFB2M leads to an increase in NAD+ levels, thereby activating SIRT3. SIRT3 is an energy sensor and deacetylase that, when activated, promotes the deacetylation modification of HIF-1α [110], enhancing its stability and activity. Activated HIF-1α further promotes HIF promotes the expression of glycolytic genes GAPDH, LDHA, GLUT1, and HK2, while downregulating PGC-1α expression to reduce mitochondrial biogenesis, enhancing the reprogramming of glucose metabolism from oxidative phosphorylation to aerobic glycolysis, thereby facilitating the progression of HCC [111].

Although the precise regulation of TFB2M in tumor progression has not been extensively studied, some research has demonstrated the potential value of modulating TFB2M expression for the treatment of cancer. Mark Ziemann et al. discovered that the tumor suppressor microRNA-101-3p (miR-101-3p) disrupts mitochondrial DNA (mtDNA) transcription by downregulating the mitochondrial transcription initiation complex proteins TFB2M and Mic60 [112]. This disruption leads to impaired mitochondrial function in osteosarcoma cells and downregulates multiple mitochondrial processes, including oxidative phosphorylation, pyruvate metabolism, the citric acid cycle, and phospholipid metabolism, ultimately inhibiting osteosarcoma cell proliferation [112]. Additionally, Cyclophilin-D (CypD) can directly interact with TFB2M to inhibit mtDNA transcription [113]. Ferezinwe and colleagues employed various MS-based proteomic approaches to investigate the interactome of Nima Related Kinases 5 (NEK5) [114]. Their study uncovered the association between NEK5 and mitochondrial proteins, such as TFAM, TFB2M, and MFN2, suggesting their potential involvement in mitochondrial maintenance, transcription, and repair processes [114].

### Role of MTERFs in cancer

There are four different subtypes of MTERFs, although MTERFs share structural similarity, their roles in mitochondria vary. Research has shown that when their functions are disrupted, it can lead to alterations in mitochondrial activity, mitochondrial damage, and the development of certain mitochondrial-related disorders [115–117]. However, the relationship between MTERFs and the regulation of oxidative phosphorylation activity, as well as their involvement in cell proliferation and tumor development, remains poorly understood. Yu Min et al. demonstrated that MTERF1 may exert its oncogenic activity by regulating mitochondrial gene expression and oxidative phosphorylation levels. MTERF1 can promote cell proliferation in HeLa cells by regulating oxidative phosphorylation activity. Overexpression of MTERF1 enhances mitochondrial gene transcription, increases oxidative phosphorylation activity, cyclin D1 expression, and promotes cell proliferation in HeLa cells. Conversely, downregulation of MTERF1 reduces ATP production, cyclin D1 expression, inhibits cell proliferation, and causes cell cycle arrest at the G0/G1 phase [118]. Subsequent studies by the same group found that MTERF1 expression was significantly higher in colon cancer tissues compared to normal colon tissues. Overexpression of MTERF1 in colorectal cancer (CRC) promotes cell proliferation, migration, invasion, and tumor formation. Additionally, MTERF1 regulates the AMPK/mTOR signaling pathway, leading to increased replication, transcription, and protein synthesis of mitochondrial DNA (mtDNA) in colorectal cancer cells. It also increases ATP levels, mitochondrial cristae density, mitochondrial membrane potential, and oxygen consumption rate (OCR), while reducing ROS production, thereby enhancing mitochondrial oxidative phosphorylation (OXPHOS) activity [13]. In recent years, studies have found that MTERF3, as an oncogene, plays a role in various cancers and is amplified and highly expressed in many different types of cancer. MTERF3 gene amplification and upregulation are negatively correlated with overall survival in cancer patients [119]. Overexpression of the MTERF3 gene promotes tumor cell growth both in vivo and in vitro, increasing the percentage of cells in the S phase [120]. MTERF3 plays an oncogenic role in colorectal cancer development by upregulating interleukin 6 and interleukin 11 to promote colorectal cancer cell growth and enhance radiotherapy resistance [121]. Furthermore, the expression of MTERF4 is essential for tumor cell proliferation, and knocking down MTERF4 in HeLa cells leads to sub-G1 cell accumulation and apoptosis [122].

## Mitochondria transcription and cancer-associate cachexia

Cachexia is a devastating, intricate metabolic syndrome characterized by significant skeletal muscle loss (accompanied by fat depletion) that occurs in around 50–80% of cancer patients and many other severe wasting diseases such as heart failure, chronic obstructive pulmonary disease (COPD), nephrotic syndrome, and AIDS [123]. The cancer-induced cachexia is associated with bad prognosis and poorer quality of life in cancer patients and the one of the main reasons contribute to cancer-related mortality [124]. Mitochondria are essential for muscle function, ensuring ATP production, metabolic adaptability, and maintenance of antioxidant defenses [125]. Recent research underscores the pivotal role of muscle mitochondria in the progression of cancer-induced cachexia [126–128] highlighting that mitochondrial dysfunction is a key driver of skeletal muscle wasting. Alterations in various mitochondrial processes observed in animal models of cancer cachexia are acknowledged to exacerbate cachexia’s development [129–131].

Mitochondrial transcription is essential for accurately expressing genes encoded by mitochondrial DNA (mtDNA), vital for oxidative phosphorylation and ATP production. Changes in this transcriptional machinery may significantly disrupt mitochondrial function, energy metabolism, and muscle homeostasis.Research on animal models of cancer-induced cachexia has revealed a decrease in mitochondrial mass and levels of mitochondrial DNA in skeletal muscle [124, 128, 132], along with a significant decrease in the expression of genes regulating mitochondrial biogenesis, such as PGC-1α and TFAM [133, 134]. This decrease is accompanied by reduced muscle oxygen consumption and ATP synthesis rate, and also downregulation of genes involved in the tricarboxylic acid (TCA) cycle [135–137]. Recent research has found that extracellular vesicles (EVs) secreted by breast cancer cells containing miR-122-5p are transferred to myocytes. In myocytes, miR-122-5p targets the tumor suppressor TP53, thereby reducing the expression of TP53 target genes involved in mitochondrial regulation, including TFAM, PGC-1 α, SCO2, and 16S rRNA. This mediates mitochondrial dysfunction in skeletal muscle during cancer and may lead to muscle weakness in some cancer patients [138].

Given the critical role of mitochondrial dysfunction in cancer-induced cachexia, some studies have attempted to alleviate cachexia by promoting mitochondrial biogenesis, achieving promising results. For instance, RYUNI et al. report that Ginsenoside Rg3 enhances mitochondrial biogenesis by promoting the activity of PGC-1α and the expression of its mitochondrial biogenetic transcription factors, nuclear respiratory factor 1(NRF1), and TFAM, effectively preventing mitochondrial dysfunction in myotubes induced by dexamethasone (DEX) and suggesting its potential in alleviating cachexia [17]. Another study showed that treatment with trimetazidine (TMZ) enhances the expression of the mitochondrial biogenesis-related transcription factors TFAM and PGC-1α in muscle cells, improving muscle mass and strength in cachectic C26-bearing mice. Similarly, direct overexpression of PGC-1α and TP53 also promotes mitochondrial biogenesis, thereby abolishing mitochondrial myopathology, enhancing endurance capacity, and improving skeletal muscle quality [138, 139].

## Conclusion and future perspectives

The advancements in structural biology, proteomics, and high-throughput sequencing technologies have greatly facilitated our understanding of the complex assembly, conformational changes, and DNA recognition mechanisms involved in key steps of mitochondrial transcription, such as initiation, elongation, and termination. However, the physiological and pathological signaling that regulates mitochondrial transcription, as well as the molecular and cellular mechanisms involved, remains limited. Further exploration of the molecular mechanisms underlying the communication between mitochondria and the nucleus in regulating mitochondrial transcription will contribute to a deeper understanding of the relationship between mitochondrial transcription dysregulation and diseases, thereby providing valuable insights for the development of drugs targeting mitochondrial transcription dysregulation for the treatment of various diseases.

It is well known that rapidly proliferating tumor cells rely on mitochondrial metabolism, with a subset of cells within the tumor tissue referred to as cancer stem cells or tumor-initiating cells heavily rely on mitochondrial metabolism, particularly the oxidative phosphorylation, for their proliferation. Tumor cells and CSCs require continuous generation of new functional mitochondria to sustain their metabolic products and energy supply, and the transcriptional regulation of mitochondrial DNA (mtDNA) plays a crucial role in the functionality of newly formed mitochondria through oxidative phosphorylation (OXPHOS). Studies have shown a close association between abnormal expression of mitochondrial transcription factors and tumor progression. This suggests that the regulation of mitochondrial transcription plays a significant role in tumor development. Dysregulation of mitochondrial transcription factors can lead to mitochondrial dysfunction, thereby affecting the metabolism, proliferation, and survival capacity of tumor cells. Therefore, understanding the role of mitochondrial transcription in cancer can have implications for cancer diagnosis, prognosis, and treatment. Targeting mitochondrial transcription or related pathways may provide potential therapeutic strategies for cancer treatment. Additionally, assessing mitochondrial transcriptional profiles or biomarkers in cancer cells or patient samples may offer diagnostic or prognostic information. Last, but not least, although not yet extensively researched, dysregulation of mitochondrial transcription may play a significant role in cancer-associated cachexia. Clinically, the judicious use of drugs that stimulate mitochondrial biogenesis could hold significant potential for alleviating cachexia.

Overall, mitochondrial transcription and its dysregulation are emerging as important factors in cancer biology, providing new insights into tumor metabolism and potential therapeutic targets. Further research is needed to elucidate the precise mechanisms and therapeutic implications of mitochondrial transcription in cancer.
